# The Transdermal Delivery of Therapeutic Cannabinoids

**DOI:** 10.3390/pharmaceutics14020438

**Published:** 2022-02-18

**Authors:** Haleh Mahmoudinoodezh, Srinivasa Reddy Telukutla, Sukhvir Kaur Bhangu, Ava Bachari, Francesca Cavalieri, Nitin Mantri

**Affiliations:** 1 The Pangenomics Lab, School of Science, RMIT University, Bundoora, VIC 3083, Australia; s3826111@student.rmit.edu.au (H.M.); srinivasareddy.telukutla@rmit.edu.au (S.R.T.); s3756626@student.rmit.edu.au (A.B.); 2 School of Science, RMIT University, Melbourne, VIC 3000, Australia; roop.bhangu@rmit.edu.au; 3 Applied Chemistry and Environmental Science, RMIT University, Melbourne, VIC 3000, Australia; francesca.cavalieri@rmit.edu.au; 4 The UWA Institute of Agriculture, The University of Western Australia, Perth, WA 6009, Australia

**Keywords:** transdermal, topical, therapeutic cannabinoids, THC, CBD, bioavailability

## Abstract

Recently, several studies have indicated an increased interest in the scientific community regarding the application of *Cannabis sativa* plants, and their extracts, for medicinal purposes. This plant of enormous medicinal potential has been legalised in an increasing number of countries globally. Due to the recent changes in therapeutic and recreational legislation, cannabis and cannabinoids are now frequently permitted for use in clinical settings. However, with their highly lipophilic features and very low aqueous solubility, cannabinoids are prone to degradation, specifically in solution, as they are light-, temperature-, and auto-oxidation-sensitive. Thus, plant-derived cannabinoids have been developed for oral, nasal-inhalation, intranasal, mucosal (sublingual and buccal), transcutaneous (transdermal), local (topical), and parenteral deliveries. Among these administrations routes, topical and transdermal products usually have a higher bioavailability rate with a prolonged steady-state plasma concentration. Additionally, these administrations have the potential to eliminate the psychotropic impacts of the drug by its diffusion into a nonreactive, dead stratum corneum. This modality avoids oral administration and, thus, the first-pass metabolism, leading to constant cannabinoid plasma levels. This review article investigates the practicality of delivering therapeutic cannabinoids via skin in accordance with existing literature.

## 1. Introduction

For many years, cannabis has been used both as a fibre source and as an edible seed [1,2]. Most notably, it produces a distinctive category of terpenophenolic compounds known as cannabinoids [2]. Cannabinoids are the principal bioactive components of this plant; however, other compounds of interest, such as terpenoids and flavonoids, have also been reported [3]. In recent years, the pharmacological characteristics of cannabinoids have been widely studied, and new applications of cannabis extracts have been proposed [4]. Due to the medicinal and recreational value of cannabinoids, cannabis agribioculture is a flourishing industry. Countries that lead investments in this marketplace include the USA, Canada, and Australia, with signifigant investments in both cultivation and manufacturing facilities [5]. 

Based on the production source, cannabinoids have been categorised into three groups: (i) phytocannabinoids; (ii) endogenous cannabinoids; and (iii) synthetic cannabinoids [6,7,8,9] (Table 1). This review mainly focuses on the plant-derived cannabinoids. Resources have reported nearly 565 cannabis constituents in *C. sativa*; 120 are phytocannabinoids, some of which have been extensively explored for their therapeutic potential. The predominant cannabinoids in plant material are delta-9-tetrahydro-cannabinol [10], cannabidiol (CBD), and cannabichromone (CBC) [4]. Table 2 depicts the significant therapeutic effects of various phytocannabinoids [11,12]. 

To date, investigations into medicinal cannabinoid applications for cancer have demonstrated significant promise, both as a supportive chemotherapeutic adjunct and from a direct anticancer perspective [12,22,23]. In the scope of palliative care, cannabinoids have demonstrated significant benefits for the management of chemo- or radiotherapy that is associated with pain, nausea and a loss of appetite [24,25]. The anticancer capacity of cannabinoids has been shown to involve multiple steps in the carcinogenesis process, including its initiation, promotion, and progression [26]. Cannabinoids act as blocking or suppressing agents in these mechanistic pathways against various cancer types, including breast, ovarian, cervical, lung, skin, colon, prostate, and brain cancers, as well as leukaemia [23,27]. Furthermore, studies since the late 1990s indicated that multiple cannabinoids, especially CBD and THC, initiate antitumour impacts in a vast range of in vitro to in vivo experiments on various cancer cells, individually, but also in combined administrations [18,28]. 

### Cannabinoid’s Receptors

The medicinal and psychoactive effects of phytocannabinoids are mediated via the endocannabinoid system (ECS) present in all tissues. In health and disease, the ECS involves several regulatory mechanisms via G protein-linked receptor-mediated signalling pathways [29]. The two famous subtypes of G protein-coupled receptors (GPCRs) are CB1, which is mainly expressed in the nervous and immunological systems, and CB2, which is incorporated in cytokine release in immune cells [30,31]. Most investigations show that ∆9-THC has an affinity to cannabinoid receptor (CBR)-dependent pathways (CB1 and CB2 receptors) [32,33]; on the other hand, non-psychoactive cannabinoids, such as CBD, regulate the activity of other deorphan and orphan G protein receptors (GPCRs) and non-GPCRs [34,35]. In 1999, GPR55 was identified as an orphan GPCR [36]. Several other orphan receptors are GPR23, GPR18, GPR120, and GPR84. The transient receptor potential (TRP) family of the cation channels are another sort of receptor that consists of six subfamilies: TRPML, TRPP, TRPC, TRPV, TRPM, and TRPA. TRPs are triggered in various stimulus transductions, including light, flavour, electrical charge, temperature, mechanical, and osmotic stimuli [37]. All types of cannabinoids may activate or suppress different members of TRPs. From the abovementioned TRP channels, five of them have been proposed to interact with cannabinoids: TRPV1 (CBD), TRPV2 (Δ9-THC, CBD, CBN), TRPV4 (CBDV, THCV, cannabigerolic acid, CBD, and CBG), TRPM8 (CBG), and TRPA1 (Δ9-THC, CBDA, CBG, and CBC) [38].

**Figure 1:**
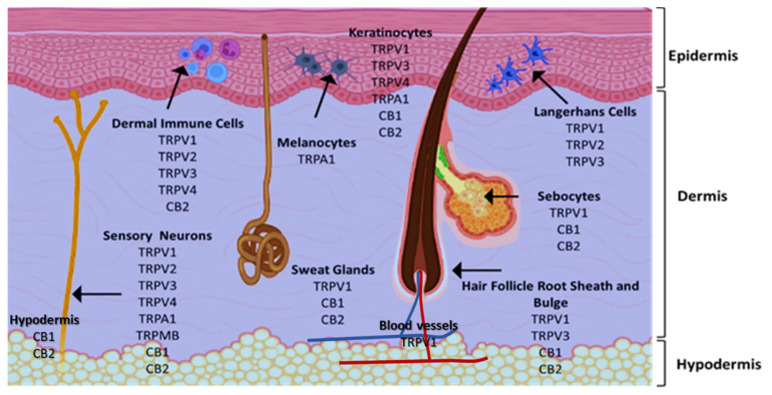
The endocannabinoid system of the skin.

Cannabinoids are lipophilic agents that bind to previously mentioned endocannabinoid receptors that regulate numerous signalling pathways in many tissues and organs, including skin, blood vessels, immune cells, lungs, liver, and the brain for the re-establishment of homeostasis following multiple disorders [25,39], for instance, pain and inflammatory management [40], Alzheimer’s disease [41], and cancer [18,27,42,43]. Figure 1 shows the cannabinoid receptor expression across skin cell types, leading to their proliferation, growth, differentiation, apoptosis, and cytokine activity [29,44,45]. CB1 is expressed in keratinocytes within the more differentiated epidermal layers, hair follicle cells, sebaceous glands, sensory neurons, melanocytes, and immune cells in human skin. CB2 is expressed in keratinocytes, sebaceous glands, sensory neurons, and immune cells. TRP channels express skin cell types [38,46,47]. The expression of all the above mentioned receptors makes the skin a tremendous potential target to deliver phytocannabinoids to treat a multitude of dermatological diseases affecting human health, e.g., eczematous eruptions, acne and seborrhoea, fibrotic skin disease, psoriasis, and skin cancer [45,48]. In terms of skin cancer, several key signalling pathways and cellular processes crucial to tumour development are targeted by endogenous cannabinoids and phytocannabinoids [39,42,49]. For example, cannabinoids promote the cell cycle arrest and apoptosis, as well as preventing proliferation, migration, and angiogenesis in cancer cells [43,49]. The impact of THC on CB1/CB2-receptor-deficient mice (Cnr1/2−/−) was evaluated. These mice were crossed to reproduce mice with a dark skin phenotype with a deficiency in CB1 and CB2 receptors. This study evaluated the effect of phytocannabinoid THC on the growth of the murine melanoma cell lines, HCmel12 and B16. THC can bind to both of the receptors. CB1 and CB2 receptors might be detected on these cell lines even with low expression levels. The THC treatment had zero impact on the cell proliferation of HCmel12 or B16 cells in vitro. In their transplantable mouse tumour model, the systemic administration of THC significantly decreased the growth of HCmel12 melanomas compared to the vehicle-treated controls. THC seems to have zero impact on mice with no CB1 and CB2 receptors (Cnr1/2−/−). Therefore, it was clear that the anti-tumour effect of THC on melanoma cells was linked to the cannabinoid receptors [50]. The presence of cannabinoid receptors in various skin cells show the value of the multiple efforts made to formulate cannabinoids to take advantage of this high potential route of delivery, due to its large surface area (nearly 20 square feet) to manage some dermatological conditions [51]. Moreover, b improving cannabinoid permeability through the skin into deeper layers and into blood circulation [52] may result in the sustained delivery of phytocannabinoids to targeted organs and tissues.

## 2. Delivery Routes and Bioavailability of Cannabinoids

The vital characteristics that impact a drug’s capacity for binding at target sites of action are its physiology; dissolution; stability; permeability; and metabolism. These factors contribute to the bioavailability of drugs, resulting in a proper or an insufficient treatment efficacy and high interindividual variability of drug efficacy in pharmacokinetic response parameters [53,54,55].. Various cannabinoids have shown similar pharmacokinetic and physicochemical properties. Furthermore, with high lipophilicity and low stability characteristics, the poor bioavailability of these agents was confirmed after some different routes of administration [53,56]. Cannabinoids demonstrated high hydrophobic specifications, with a log *p*-value ranging from 6 to 7, with a slight miscibility in water (2–5 µg/mL). They are significantly light- and temperature-sensitive. Mazzetti et al. demonstrated that 13% of CBD samples kept at ambient temperatures succumb to auto-oxidation within 30 days; this was especially prevalent in solutions and in exposure to light [15,57,58]. Thus, the plant-derived cannabinoids require enviro-protective engineering solutions to reduce the degradation of the bioactive components during the storage and administration of these drugs to patients. Several such solutions have been developed for oral, nasal-inhalation, intranasal, mucosal (sublingual and buccal), transcutaneous (transdermal), local (topical), and parenteral routes of drug delivery [56].

On the other hand, the bioavailability, pharmacokinetics, time course, and efficacy of cannabinoids differ significantly depending on the methodology of administration [15,53]. The most explored administration route, the oral delivery of cannabinoids, has significant drawbacks due to the extensive inactivation via the hepatic first-pass metabolism (over 95%), and its slow absorption, which, remarkably, affects the overall therapeutic efficacy [59,60]. Additionally, the time course duration and the subsequent difficulty predicting the administrative dosage further contributes to oral administration’s inefficacy. Systematic bioavailability data for the oral administration of THC and CBD have been reported at 10 to 20% [35] and 6 to 19% [53,60,61], respectively. The plasma concentration of CBD was found to peak 1 to 4 h post-administration in a dose-dependent manner, and decreased significantly after 6 h [35,55,56]. Additionally, the oral administration of CBD has been shown to degrade into THC [10] and other psychoactive cannabinoids when exposed to a highly acidic stomach environment [62]. In a dog model study, the pharmacokinetics of CBD were assessed for oral administration; the hepatic metabolism of CBD was implicated in a lack of detected CBD in blood plasma [60]. Following oral administration for cancer therapy, absorption may be limited by CBD degradation by the stomach acids and hepatic influences, leading to frequent dosing requirements, which are often not feasible in nauseated patients [56,63]. The bioavailability of cannabinoids through smoking has previously been reported to be between 2 and 56%, due to a variability in smoking dynamics, which causes uncertainty in dosage delivery [64,65]. The vaporisation of CBD was found to reach blood plasma concentrations of up to 50% [59,60]. During THC’s inhaled administration, the highest time course was evident after a duration of 3 to 10 min, and this effect was shown to diminish in a dose-dependent response between 4 to 12 h [66,67]. Inhaled cannabinoids expose valid concerns about the unfavourable pulmonary impacts and the limited effectiveness when the intended impact is not well-targeted [56,68]. Additionally, the model with which cannabinoids are inhaled has dramatically variable effects depending on the length and the volume of inhalation, the held inhalation duration, and rate at which these inhalations occur [56,69].

Other methods of cannabinoid administration include intranasal, rectal, and intravenous [15,70]. The rectal administration of THC demonstrated approximately doubled rates of bioavailability compared to oral routes. This is attributed to reduced rates of hepatic enzyme metabolism, the lack of acidic degradation, and higher absorption via rectal tissues [71]. Intranasal administration has been assessed for a variety of cannabinoid formulations. Due to the high prevalence of vascular structures in the nasal cavity, absorption is rapid [15], and blood plasma levels achieve a moderate bioavailability range of 34–46% [72]. Additionally, nasal administration circumvents the hepatic degradation of cannabinoids, avoiding this metabolic hurdle [15,72,73]. Intravenous administration is challenging due to the poor water solubility of cannabinoids [56]. Moreover, injection-based drug administration is undesirable due to its invasive nature, the increased risk of infection, and the lack of compliance in patients [15]. The topical and transdermal application of cannabinoid products has shown higher bioavailability rates in the presence of enhancers and has also prolonged steady plasma concentration compared to other routes of delivery [72,74,75]. Additionally, the acidic degradation of CBD to THC, with the consequent psychotropic effects, is effectively mediated via its transdermal application due to the exclusion of digestive processes and a neutral skin environment [62]. Transdermal patches imbued with Δ8-THC induced a steady-state plasma concentration within 1.4 h post-administration, which was preserved in subjects for at least 48 h [63]. In a murine model, CBD was applied via an ethosomal system transcutaneously; this treatment resulted in steady CBD plasma levels for 72 h, suggesting that this is a promising future delivery method for cannabinoids in a clinical setting [52,63]. In terms of the cannabinoids’ preferred prescribing route, topical administration has additional promises in its prescribability. A systematic review published by Yeroushalmi et al. performed a pilot survey to determine the willingness of dermatologists to recommend medical cannabis, and 75% of dermatologists recommended topical formulations [76]. This route is likely preferred due to its convenience, without the concerns of the psychoactive impacts of systemic absorption, as well as the high safety profile of topical administration routes [77]. 

## 3. Transdermal Delivery of Cannabinoids

### 3.1. Skin as a Potential Route

Skin is the largest organ of the human body. In terms of its immunological function, it is a barrier between the body and the neighbouring environment [78,79], consisting of three layers: dermis, epidermis and Stratum corneum (SC) [80,81]. Skin unique characteristics make it permeable to the surrounding environment and allow the diffusion of air, heat, fluids and low molecular weight molecules [78]. Skin diffusion can occur by (a) the intercellular way, through the gaps between the corneocytes or (b) the transcellular way, through the corneocytes and neighbouring lipid matrix or (c) the appendageal way; via the sweat glands and hair follicles [82]. This attribute can be used as an alternative means for drug delivery, especially for transdermal application into the blood circulation, and it is easier for patience than oral and parenteral administration [51,83]. Transdermal administration is mainly to effect locally, where it can (i) get rid of the necessity for systematic drug therapies, (ii) lessen the total dosage needed to reach the targeted site, and (iii) decrease side effects [84].

### 3.2. Importance of Transdermal Drug Delivery Studies

Transdermal drug delivery systems (TDDS) have gained significant attention recently due to a vast range of advantages linked to self-administration, preferred patient compliance, bypassing of the first-pass effect, and immune-surveillance functions [85,86]. TDDS can increase medical efficacy and reduce adverse effects, especially cancer treatment [87,88]. It can efficiently avoid poor absorption caused by gastrointestinal pH and first-pass metabolism, maintaining a continuous and lengthy drug plasma concentration [89,90], improving patient compliance and reducing side effects [85,91]. Additionally, drug therapy may be aborted abruptly by dismissing the application from the skin’s surface [92]. 

### 3.3. Transdermal Delivery of Cannabinoids and Challenges

All advantages mentioned above of transdermal drug delivery over other formulations and considering dermatologists are interested in recommending topical formulations of cannabinoids to patients [93]. In addition, different ways of transcutaneous formulations are promising to produce local pharmacological and systematical effects [94].

On the other hand, some criteria should be considered to have successful transdermal delivery in terms of drug physicochemical properties. FDA-approved molecules for transcutaneous drug delivery are those with low molecular weight (<500 Da) with balanced lipophilicity and hydrophilicity balance (log P = 1–3) as transdermal drug delivery systems require to pass through the hydrophobic Stratum corneum lipid matrix and followed by absorption into the deeper aqueous layers of skin and the systemic circulation [51,95]. Despite the desired range of molecular weight of cannabinoids, with log p between 6–7 and low water solubility, their transdermal delivery is a challenge. Thus, enhancing the cannabinoid permeation via the skin has been performed by applying different strategies such as the use of chemical penetration enhancers (oleic acid, ethanol, methanol leading to improve cannabinoids diffusion through the skin), microemulsions, physical enhancer (including microneedles, electroporation iontophoresis, ultrasound, magnetophoresis to gain proper levels of skin permeation), encapsulation in micro/nano gels, nanoparticles and nano-carriers [15,45,51]. Additionally, the potential promising benefits of applying nanoformulation and nanoencapsulation systems can be improving the effective doses delivery of highly lipophilic drugs (e.g., cannabinoids), protecting poor stable therapeutic agents from aggressive environments, and targeted and controlled delivery [96]. Hence, considering cannabinoids physicochemical limitations, they may benefit from nanotechnology approaches to overcome the Stratum corneum barrier [97,98]. 

Table 3 depicts several reports of the transcutaneous application of cannabinoids for various purposes like reaching a steady-state plasma cannabinoids concentration by using patches and gels, applying different techniques to enhance cannabinoids permeability through the skin, and also devising different formulations to encapsulate these therapeutic agents to improve physicochemical properties and penetration via skin layers. 

Sustained drug delivery benefits of TDDS provides a steady-plasma concentration of therapeutic agents (especially with limited half-life) and not cause of peaks in plasma levels compared to other delivery routes of [74] cannabinoids, like 1.4–10 h and 24 h after oromucosal spray and intravenous administration of CBD, respectively [55]. In vitro and in vivo permeability studies of Δ8-THC, an isomer of Δ9-THC with lower psychotropic side effects in hairless guinea pig skin and human skin was tested [99,100]. This isomer has been administrated to cancer patients before chemotherapy to control vomiting [99]. Two relevant results were reported; First, the diffusion ratio of Δ8-THC in guinea pig and human skin was nearly the same regardless of membrane composite in vitro. Second, applying the transdermal patch revealed Δ8-THC concentrations increased gradually in the plasma and perceived an average steady-state plasma concentration of 4.4 ng/mL for more than 48 h. In addition, the steady-state levels did not change after removing the patch for another 24 h [101]. 

Significant cumulation of the CBD in the skin (a murine model) was reported when a patch contained ethosomal systems containing CBD (40% *w*/*w* ethanol, 3% *w*/*w* CBD in a carbomer gel), with 200–400 nm in diameter, used to chronic inflammatory diseases. 24 h after administration of the formulated ethosomal CBD to the skin of nude mice with rheumatoid Arthritis (RA) showed a significant CBD accumulation not only in the abdominal skin, abdominal muscle and hip skin but also in the liver, pancreas and hip muscle. Furthermore, the CBD plasma concentration after 12 h was around 23% of the initial dose, and the steady-state CBD plasma levels reached about 24 h and lasted for 72 h (~44% of initial dose). This study proved that the CBD was delivered efficiently to the inflammatory organ through ethosomal carriers and able to maintain the therapeutically steady-state levels at the site [52].

The protective effect of CBD (gel formulation) for alcohol-induced neurodegeneration was investigated in a rodent model [102]. Two experiments were devised to evaluate the neuroprotection of the CBD gels. The 5.0% CBD gel in the first experiment and 2.5% CBD gel in the second experiment resulted in a 48.8% and 56.1% of reduction in neurodegeneration in the entorhinal cortex. Furthermore, 2.5 g CBD formulated per 100g gel (containing Transcutol® HP as a permeation enhancer) proofread the medicinal advantages of cannabinoids in preventing relapse to drug use in a rat model. In another study, the anti-relapse effect of transdermal CBD formulation was investigated in rat models of anxiety, drug seeking and impulsivity. In this study, CBD was administered to rats with histories of cocaine or alcohol self-administration for seven days with 24 h intervals and was observed for experimental anxiety and stress-induced reinstatement. Transdermal application of CBD showed anti-anxiety activity in rats with cocaine and alcohol dependence history and prevented the devolvement of impulsivity in rats with alcohol dependence histories. Interestingly, after the termination of treatment, reinstatement remained reduced for five months, although concentrations of CBD in the brain and plasma were detectable for only three days [20].

Another study illustrated that the transcutaneous delivery of CBD via gel (0.6, 3.1, 6.2 or 62.3 mg/day) administered four consecutive days onto the skin resulted in a significant reduction of proinflammatory markers (CGRP, OX42 and TNFα) joint swelling and immune infiltration in a rat model of Arthritis and thickening of the synovial membrane in a rat model of Arthritis without psychoactive side-effects. With long-lasting therapeutic effects and without apparent adverse effects, transdermal gel with 6.2 and 62 mg/day doses of CBD indicated effective doses with long-lasting therapeutic effects. Furthermore, the linear plasma CBD concentration was revealed in three lower doses [40]. 

Moreover, these results closely resemble another in vitro experiment performed on rat skin using oleic as a permeation enhancer in a transdermal formulation containing THC. Application of formulation to the rat skin resulted in steady plasma concentrations for about 24 h, suggesting a sustained THC delivery to the bloodstream [103]. Hence, remaining net cannabinoids molecules at a steady level in plasma for prolonged periods confirmed the advantages of transdermal delivery as a potential route of consistent delivery of efficient cannabinoids doses needs to be studied more.

The topical administration of CBD ointment improved the quality of life in patients with skin problems (psoriasis, dermatitis and scars) [19]. Interestingly, a hydrophilic gel with 79% *w/w* propylene glycol showed the best performance for topical administration of CBD [104].

As is summarised in Table 3, to improve physicochemical properties and the skin permeability of cannabinoids, various methods were proposed to encapsulate phytocannabinoids for different purposes. THCA and CBDA were specifically studied recently to formulate a stable microemulsion (a nanosized drug delivery system) to deliver them transdermally, and permeation of cannabinoids-loaded microemulsion was also examined concerning pH. Results confirmed formulation with 1.0% (*w*/*w*) of cannabinoids showed a significant improvement of THCA and CBDA permeation rates and amounts at pH values of 5.17 (17.13-folds increase) and 5.25 (11.63-folds increase), respectively in in vitro skin models. This report shows that after six months of storing cannabinoids-loaded microemulsion at 4 ℃ and 25 ℃, over 95% stable acidic cannabinoids content was maintained, indicating microemulsion system is a promising strategy for improving the stability and permeability of cannabinoids [105].

For controlled transdermal delivery of CBD, patches based on stimuli-responsive chitosan and Zinc oxide [106] nanoparticles are developed and tested on L929 mouse fibroblasts [107]. The results showed higher drug loading efficiencies, the prolonged release of CBD within six days, and greater biocompatibility [106]. A clinical trial study on 48 patients aged 3–18 years demonstrated that transdermal administration of a CBD-loaded gel was safe, well-tolerated during the 6.5 months treatment period. In addition, reduction in complex partial seizures (FIAS) and tonic-clonic seizures (TCS) frequency by 44.5% and 22.7%, respectively, were observed, resulting in an improved quality of life [108].

A super-macroporous cryogels containing CBD was fabricated using 2-hydroxyethyl cellulose and β-cyclodextrin for the treatment of various skin malignancies. The fabricated matrixes showed high drug encapsulation efficiency (100%), with bi-phasic release profiles (immediate release within 3 h followed by sustained CBD release over 24 h. The formulated CBD showed concentration-dependent cytotoxicity against two human tumour cell lines, MJ (cutaneous T-cell lymphoma) and HUT-78 (Sezary Syndrome) [109]. In another study by this group, CBD-loaded polymeric micelles were embedded into biodegradable 2-hydroxyethyl cellulose (HEC), which showed sustained CBD released profiles in vitro and preserved the anticancer activity the drug [110].

Other recent findings on using eco-friendly and surfactant-free techniques of applying Pickering emulsion stabilised by chitosan/collagen peptide (CH/CP) nanoparticles loaded with CBD demonstrated significant long-lasting stability after five months with high CBD content (99.45% of the initial loaded amount) and also non-toxic to skin keratinocytes [111]. Furthermore, an ex-vivo skin study on Porcine skin samples confirmed that after 24 h of topical administration, these CBD-loaded CH/CP nanoparticles had low permeability into the deeper layers of skin, and CBD was kept in high concentration in the *Stratum corneum*. Thus, stabilising the biocompatible and biodegradable CH/CP nanoparticles by Pickering emulsions overcome the *Stratum corneum* barrier and make this green developed vehicle suitable for topical the highly lipophilic unstable CBD [112].

The potential of CBD-loaded oleic acid microemulsion formulated as microemulgel was investigated to treat various cutaneous diseases, including psoriasis, eczema, pruritus, and inflammatory conditions. Encapsulation of CBD in microemulgel improved the solubility and stability of CBD, with a controlled release profile over 24 h in vitro. The formulation demonstrated suitable viscosity with good skin adherence. In addition, the permeability of CBD through rabbit ear skin showed good retention in the skin layers indicating the great potential of this carrier for topical delivery of CBD [113].

**Table 3:** Some studies on cutaneous delivery of cannabinoids.

Citations	Formulation	Active Substance	Concentrate	Applications	Result
[19]	Ointment	Cannabidiol (CBD)	-	Inflammatory skin diseases and cutaneous scars (psoriasis, atopic dermatitis, and scars)	Improved skin parameters such as hydration, transepidermal water loss, and elasticity in humans
[20]	Hydroalcoholic proprietary gel	Cannabidiol (CBD)	2.5 g CBD/100 g gel permeation enhancer (Transcutol^®^ HP)	The prevention of relapse to drug use (alcohol or cocaine)	CBD has potential in relapse prevention in the rat model
[40]	Gel	Cannabidiol (CBD)	6.2 and 62 mg/day	Inflammation and pain	Reduction of proinflammatory markers, joint swelling, and immune infiltration in rat model of arthritis
[63]	Patches	Δ8-THC	16 mg/mL D8 -THC in 1:1:1 (*v*/*v*/*v*) of propylene glycol:water:ethanol	Making a transcutaneous drug system (TTS) for Δ8-THC	Gradually enhanced Δ8-THC plasma concentration and preserved steady-state plasma levels 24 h after removing the patch
[52]	Patches	Cannabidiol (CBD)	3% *w*/*w* CBD and 40% *w*/*w* EtOH	Chronic inflammatory diseases	Significant cumulation of CBD in the skin of murine models
[102]	Gel	Cannabidiol (CBD)	The 1%, 2.5%, and 5% (*w*/*w*) CBD gels	Alcohol-induced neurodegeneration	Neuroprotection and reduction of alcohol-induced neurodegeneration in rodent models
[105]	Microemulsion	Acidic cannabinoids (THCA and CBDA)	1.0% (*w*/*w*) of cannabinoids, 5% (*w*/*w*) of Capryol^®^ 90, 44% (*w*/*w*) Surfactant mixture (2:1, Procetyl^®^ AWS and Ethanol) and 50.0% (*w*/*w*) distilled water	Permeation enhancement	A significant improvement of the permeation of THCA and CBDA in vitro.
[106]	Stimuli-responsive chitosan/ZnO NPs	CBD	-	Treatment-resistant epilepsy patient	Carrier showed superior drug loading capacity with the prolonged release of CBD for six days and great biosafety tforL929 mouse fibroblasts (the connective tissue cells)
[109]	Polysaccharide cryogels containing β-cyclodextrin	CBD	-	Skin malignancies	The designed platform showed a high drug loading efficiency with biocompatibility and bi-phasic drug release with an initial burst and a later slow release. Showed dose-dependent cytotoxicity on two cancer cell lines (MJ and HUT-78)
[110]	Nanocomposite HEC/PM cryogel	CBD	-	Cutaneous lesions due to CTCL (cutaneous T-cell lymphoma)	Sustained topical CBD delivery
[112]	Emulsions stabilized with chitosan/collagen peptides nanoparticles	CBD	0.6 g CBD in olive oil and liquid paraffin mixture to make 6 mg/mL	Cosmetic purposes	Effective penetration of nanoparticles through deeper skin layers
[113]	Microemulgel	CBD	1% *w*/*w* CBD Solutol HS 15 (20%, surfactant), Transcutol P (9%, cosolvent), isopropyl myristate (5%, oil phase), water (66%)	Skin diseases	Highly stable formulation, controlled drug release, retention in the skin layers
[114]	Prodrug (D9-tetrahydrocannabinol amino acid dicarboxylate prodrug)	THC analogue	-	Glaucoma treatment	Reduced intraocular pressure
[115]	Topical CBD (oil, cream, and spray)	Cannabidiol (CBD)		Epidermolysis bullosa	Decrease in pain and blistering; fast wound healing; no effects reported
[116]	Topical MC oil	CBD and THC	THC 5 mg/mL CBD 6 mg/mL	Patients with pyoderma gangrenosum	Improve pain management and improve baseline pain levels
[117]	Cream	Cannabidiol (CBD)	CBD-infused oil (75 mg/mL or 150 mg/mL)	Pharmacokinetics	Probable incomplete transdermal absorption in healthy dogs

## 4. Conclusions

Considering the several advantages of topical/transdermal cannabinoids, including the fact that it is the most preferred administration route among dermatologists and patients, there is much room for improvement in the transcutaneous delivery of therapeutic cannabinoids with high lipophilicity and low bioavailability features. The aqueous layers of the skin’s tissue beneath the stratum corneum present a rate-limiting step for hydrophobic cannabinoid diffusion [118,119]. Thus, in recent years, several nano-systems were proposed for the topical- and systemic-controlled delivery of cannabinoids, including micellar, liposomal, and nanosized formulations [120], as well as microemulgel [113], nano-emulsions [105,113], dendrimers, and polymeric nanoparticles [96,109,110,112]. Nano-conjugation or nano-encapsulation, as passive strategies, showed a promising potential in the transdermal delivery of cannabinoids to enhance their bioavailability, safety, stability, efficacy, and also to avoid the fluctuation of plasma cannabinoid concentrations during the treatment period, which is encountered in conventional routs of delivery [52,63,121,122]. Furthermore, physical enhancers, such as microneedles, showed a promising ability to deliver formulated lipophilic drugs to action sites [123]. Hence, more studies on the application of microneedle platforms, to enhance the efficacy of cannabinoids’ transcutaneous delivery in future, would be valuable. In conclusion, transdermal delivery products continue to be of real therapeutic benefit to patients worldwide, with a positive trend that is expected to continue in the foreseeable future [124]. In terms of the transdermal cannabinoid delivery system, a broader scope of knowledge is needed due to the limited published studies on this topic.

## Figures and Tables

**Table 1:** Cannabinoid categories.

Cannabinoids	Compounds
Plant-derived cannabinoids (phytocannabinoids)	THC, CBC, CBD, CBG, CBDV, THCV, THCAV, Δ-8-THC
Endocannabinoids	AEA, 2-AG, PEA, O-AEA, 2-AGE, 9-Octadecenamide
Synthetic cannabinoids	JWH-015, Dronabinol, Nabilone, WIN-55, 212-2, Rimonabant, CP55940, ACEA, Hu-308, AjA, (R)-methanandamide (MET)

**Table 2:** The therapeutic effects of phytocannabinoids.

Phytocannabinoids	Therapeutic Effects	Citations
THC 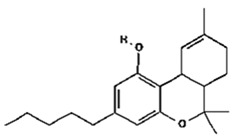	Modulation of pain, spasticity, sedation, and mood; Bronchodilator; Neuroprotective and antioxidant; Antipruritic in cholestatic jaundice; Anti-inflammatory (power: 20× aspirin and 2× hydrocortisone); Analgesic; Anti-nausea/emesis (which is induced by chemotherapy); Appetite promoter Antitumorogenic	[10,11,12,13,14,15,16,17,18]
CBD 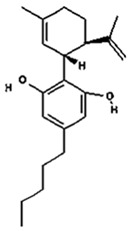	Anticonvulsive; Anti-inflammatory and immunosuppressive (psoriasis, atopic dermatitis, and abrasions); Neuroprotective and antioxidative; Antipsychotic; Counteracts the intoxicating effects of cannabis; Anxiolytic; Addiction treatment; Antimicrobial (Gram-positives bacterial cutaneous infections); Antitumour	[11,12,14,17,18,19,20,21]
CBG 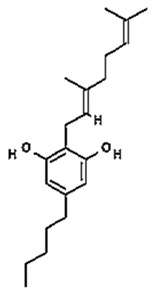	Muscle relaxant; Analgesic; Antifungal (modest); Antineoplastic; Antidepressant; Inhibition of keratinocyte proliferation in psoriasis; Antibiotic activity against methicillin-resistant *Staphylococcus aureus* (MRSA);	[11,12,14,17,18,21]
CBC 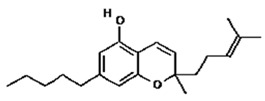	Analgesic; Anti-inflammatory	[8,9,13]
CBN 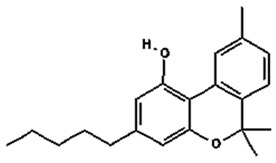	Sedative; Anticonvulsive; Anti-inflammatory; Antibiotic (with anti-MRSA activity); Inhibition of keratinocyte proliferation; Osteogensis promoting	[11,12,14]
THCV 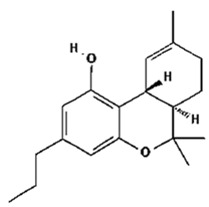	Promotion of weight loss; Anticonvulsive; Suppression of hyperalgesia and inflammation; Appetite suppression; Counteracts the intoxicating effects of THC	[11,12,14]
THCA-A 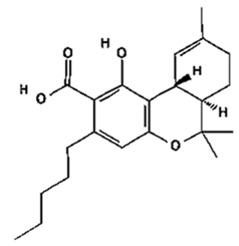	Immunomodulatory; Anti-inflammatory; Neuroprotective; Antineoplastic	[11,12,14]
CBDV 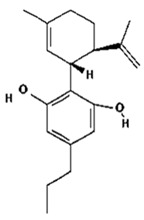	Anticonvulsant	[11,12,14]

## Data Availability

Not applicable.

## Abbreviations

2-AGE	2-arachidonylglyceryl ether (Noladin ether)
2-AG	2-arachidonoylglycerol
AEA	Anandamide (*N*-arachidonoylethanolamine)
AjA	Ajulemic acid
CBC	Cannabichromene
CBD	Cannabidiol
CBDV	Cannabigerovarin
CBG	Cannabigerol
CBN	Cannabinol
MET	(R)-methanandamid
MRSA	Methicillin-resistant *Staphylococcus aureus*
O-AEA	O-arachidonoyl ethanolamine
PEA	Palmidrol
THC	Delta-9-tetrahydrocannabinol
THCA-A	Tetrahydrocannabinolic acid
THCVA	Delta 9—tetrahydrocannabivarin carboxylic acid
THCV	Delta 9—tetrahydrocannabivarin
TRPA	Transient receptor potential Ankyrin
TRPC	Transient receptor potential canonical
TRPM	Transient receptor potential melastatin
TRPML	Transient receptor potential mucolipin
TRPP	Transient receptor potential polycystin
TRPV	Transient receptor potential vanilloid
Δ-8-THC	Delta-8-tetrahydrocannabinol
